# MRI-based assessment for optimal breast cancer local therapy in the era of BI-RADS v2025

**DOI:** 10.1007/s11604-026-02033-2

**Published:** 2026-06-15

**Authors:** Maya Honda, Masako Kataoka, Mami Iima, Azusa Nishimoto, Yuji Nakamoto

**Affiliations:** 1 Preemptive Medicine and Lifestyle-related Disease Research Center, Kyoto University Hospital, 53 Kawahara-cho, Shogoin, Sakyo-ku, Kyoto, 606-8507 Japan; 2 Department of Fundamental Development for Advanced Low Invasive Diagnostic Imaging, Nagoya University Graduate School of Medicine, Nagoya, Japan; 3 Department of Diagnostic Imaging and Nuclear Medicine, Kyoto University Graduate School of Medicine, Kyoto, Japan

**Keywords:** Breast neoplasms, Magnetic resonance imaging, Mastectomy, subcutaneous, Mastectomy, skin-sparing, Lymph nodes, Breast Imaging Reporting and Data System

## Abstract

Preoperative breast magnetic resonance imaging (MRI) is the most sensitive modality for assessing disease extent in newly diagnosed breast cancer, detecting additional ipsilateral disease in approximately 20% of patients. While its impact on patients’ prognosis is debated, MRI may refine surgical management when it is appropriately evaluated. This review provides a practical framework for optimizing MRI interpretation for individualized surgical planning, incorporating the updated American College of Radiology Breast Imaging Reporting and Data System (BI-RADS) v2025 recommendations. Key updates in BI-RADS v2025 include the introduction of “Additional Close Findings” to streamline the management of suspicious lesions within 2.0 cm of the index cancer. For oncoplastic procedures, MRI provides critical metrics: a tumor-nipple distance of > 1.0 cm has been reported as a significant threshold under specific study conditions for predicting nipple involvement in nipple-sparing mastectomy, while an MRI-to-skin distance of 5 mm is a suggested threshold for predicting margin positivity in skin-sparing mastectomy. This review also addresses the assessment of pectoralis muscle involvement and the new BI-RADS section dedicated to lymph node evaluation. Furthermore, MRI is highlighted as an essential tool for identifying eligible candidates for radiofrequency ablation by precisely excluding multifocality and extensive intraductal components to satisfy strict clinical criteria. The integration of these standardized, surgically-relevant metrics allows radiologists to provide a detailed anatomical roadmap, which is essential to maximize cosmetic outcomes through conservative mastectomy techniques without compromising oncological safety.

## Introduction

Preoperative breast magnetic resonance imaging (MRI) is widely recognized as the most sensitive imaging modality for assessing the extent of disease in newly diagnosed breast cancer, primarily because of its ability to identify mammographically and clinically occult malignancies. Meta-analysis data indicate that MRI detects additional ipsilateral disease in approximately 20% of patients and contralateral disease in 5% [1]. However, its routine clinical use remains a subject of ongoing debate. While MRI offers superior cancer detection, randomized controlled trials and meta-analyses have yet to consistently demonstrate improved long-term outcomes, such as reduced recurrence rates or increased overall survival [2]. For example, the comparative effectiveness of MRI in breast cancer (COMICE), MR mammography of nonpalpable breast tumours (MONET) and the IRCIS trial, a randomized phase III study of preoperative breast MRI in women with local DCIS (NCT01112254), demonstrated that the addition of preoperative MRI did not lead to a reduction in re-excision rates for patients undergoing breast-conserving surgery (BCS) [3–5]. Notably, the MONET trial reported a paradoxical increase in the re-excision rate in the MRI group [4], while the Preoperative MRI of the Breast (POMB) trial found decrease in the re-excision rate and slight increase in 10-year survival in the MRI group [6, 7]. More recently, the large-scale prospective Multicenter International Prospective Analysis (MIPA) study confirmed that while MRI is associated with an increased rate of mastectomy, while it result in a lower rate of re-operation [8]. Despite these controversies, several studies highlight MRI’s utility in refining surgical management. MRI estimates the final pathological extent of disease more accurately compared with conventional imaging, particularly in cases of invasive lobular carcinoma and ductal carcinoma in situ [9, 10].Indeed, A patient-matched analysis from the MIPA study demonstrated a reduction in reoperations for both invasive lobular carcinoma [11] and ductal carcinoma in situ following preoperative MRI [12], though this effect was not statistically significant in the latter. Reflecting these clinical data, current international guidelines advocate for a selective rather than routine use of preoperative MRI. The National Comprehensive Cancer Network (NCCN) and the American Society of Breast Surgeons (ASBrS) both categorize preoperative MRI as an optional or non-routine tool, citing concerns over high false-positive rates and an associated increase in mastectomy rates without proven improvements in overall survival or re-excision frequency [13, 14]. Despite this cautious approach, there is broad consensus across the NCCN, ASBrS, and the European Society of Breast Imaging (EUSOBI) regarding its utility in specific scenarios, such as evaluating disease extent in invasive lobular carcinoma [13–15]. While the EUSOBI guideline more proactively recommends MRI for contralateral breast screening in all biopsy-proven cases, all three organizations emphasize that surgical decisions should not be based solely on MRI findings due to its limited specificity, necessitating histological confirmation of any suspicious indeterminate lesions [13–15].

Modern surgical management of breast cancer has evolved beyond the binary choice between breast-conserving surgery and conventional total mastectomy, increasingly incorporating “conservative mastectomy” techniques, such as skin-sparing mastectomy (SSM) and nipple-sparing mastectomy (NSM) followed by immediate reconstruction. SSM involves the preservation of the entire skin envelope while removing the nipple-areolar complex, whereas NSM preserves both the skin and the nipple-areolar complex offering superior cosmetic outcomes and higher patient satisfaction. Moreover, minimally invasive ablation therapy is increasingly recognized as promising non-surgical approaches to local tumor control. The success of these oncoplastic procedures depends on the accurate exclusion of occult tumor involvement in the preserved tissues to minimize the risk of local recurrence. Consequently, the necessity for preoperative breast MRI has become increasingly important in determining the suitability of these treatment options.

The clinical impact of preoperative MRI is influenced by factors beyond imaging technology itself. These include institutional capability for MRI-guided biopsy, and the preferences of both surgeons and patients regarding surgical approaches. Radiologists’ expertise in interpreting breast MRI also significantly affects MRI performance.

In this review, we discuss standardized interpretation methods for contrast-enhanced breast MRI, incorporating recommendations from the recently updated American College of Radiology Breast Imaging Reporting and Data System (BI-RADS) v2025 [16]. By synthesizing recent evidence with the updated BI-RADS v2025, this review aimed to provide a practical framework for optimizing MRI interpretation to better guide individualized surgical planning. The key evaluation items and their clinical implications discussed in this review are summarized in Table 1.

**Table 1:** Key preoperative MRI fi ndings for breast cancer local therapy planning

Evaluation item	Key measurement/finding	Clinical implication
Location	Clock face position/Quadrant; Depth; Distance from the nipple	Tumor localization
Size	Reported in three-dimensions	Tumor staging
	Tumor size ≤1.5 cm (or ≤ 1.0 cm depending on guidelines)	Feasible for Radiofrequency ablation
Extent	Additional Close Findings (ACFs) (≤ 2.0 cm from index lesion Total size difference ≤ 2.0 cm)	Resectable within a single excision volume
Tumor-nipple distance	≥ 1.0 cm	Feasible for nipple-sparing mastectomy
Tumor-skin distance	≥ 5 mm	Feasible for skin-sparing mastectomy
Pectoralis muscle involvement	Enhancement extending into the muscle	Requires muscle resection
	Abutment without invasion	Necessity of muscle resection is equivocal
Lymph nodes	Intramammary, axillary (level I-Ⅲ) and internal mammary nodes	Nodal staging

## Methods

This manuscript was prepared as an invited narrative review. Relevant publications were identified through targeted PubMed searches and manual review of the reference lists of key articles, using keywords such as “breast MRI”, “preoperative”, “skin-sparing”, “nipple-sparing” and “pectoralis muscle”. A formal systematic review methodology was not used; no predefined review protocol, fixed search timeframe, or exhaustive search strategy was established in advance. The review focused on studies published from 2010 onward regarding the use of contrast-enhanced MRI for breast cancer evaluation. Articles primarily addressing the assessment of response to neoadjuvant therapy were not included, as this topic falls outside the scope of this review.

## Evaluation of disease location and extent

Breast conserving surgery, typically performed in conjunction with adjuvant radiotherapy, is established as a standard-of-care locoregional treatment for early-stage breast cancer, offering overall survival rates comparable to those of mastectomy [13]. The oncologic success of breast conserving surgery is heavily predicated on achieving pathologically negative surgical margins—defined as “no ink on tumor” for invasive carcinoma—to minimize the risk of ipsilateral breast tumor recurrence [13]. However, accurately delineating the true spatial boundaries of a malignancy remains a significant clinical challenge, as conventional imaging modalities such as mammography and ultrasound might underestimate disease extent or overlook occult multifocal or multicentric disease. Consequently, MRI can be utilized to more accurately characterize the local disease burden prior to surgery, leveraging its superior sensitivity compared to conventional imaging.

**Fig. 1:**
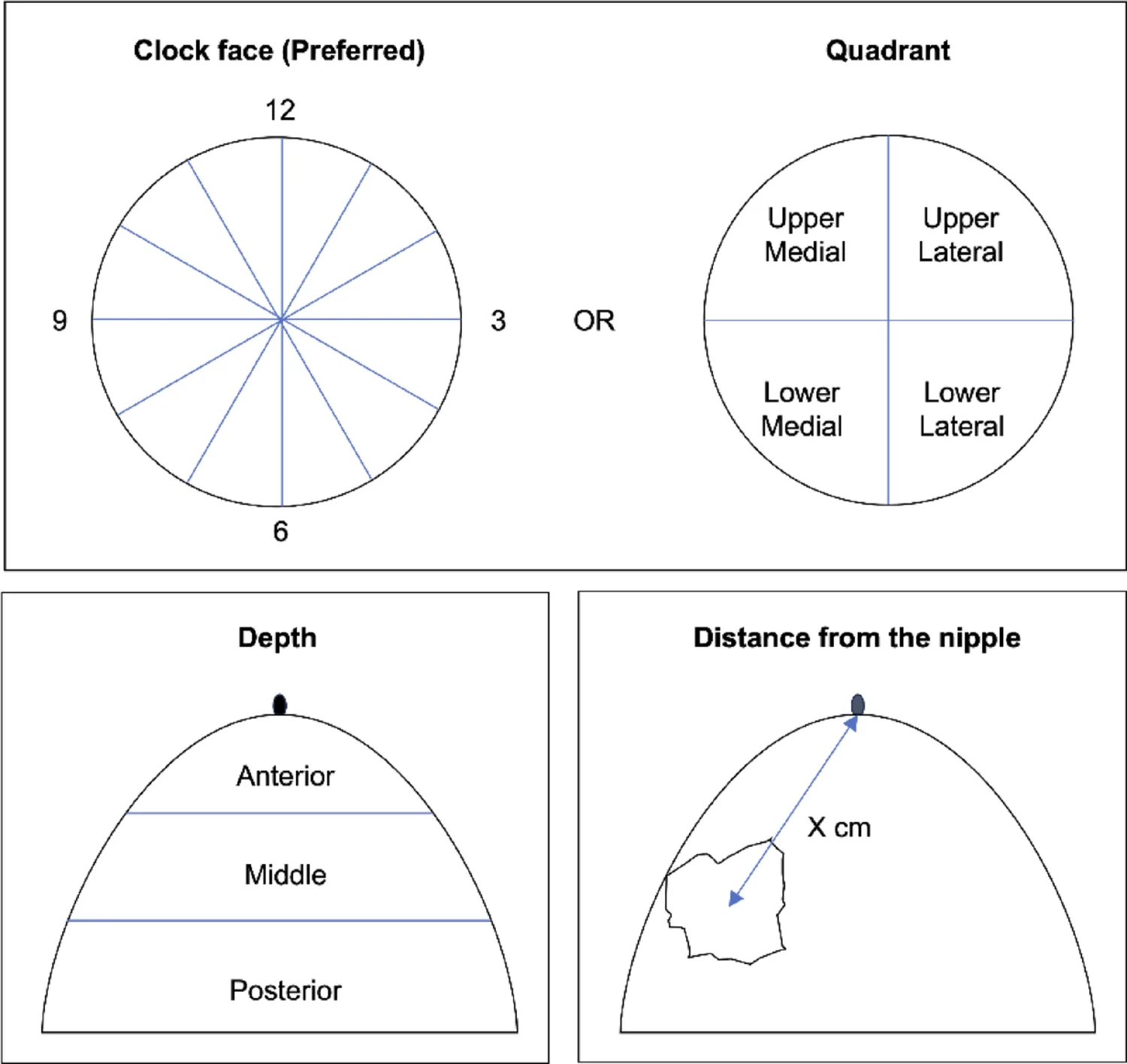
Schematic for describing lesion location

Precise localization of the lesion is paramount, particularly because of the marked positional shift of the breast tissue between the MRI (prone) and the surgical procedure (supine). To bridge this discrepancy, a multi-parameter approach to description is required. In accordance with BI-RADS v2025, lesion location should be documented using the clock face position or quadrant, with depth descriptors (anterior, middle, or posterior) and distance from the nipple [16]. The distance from the nipple is measured from its base to the center of the lesion and is described in whole centimeters (Fig. 1) [16].

**Fig. 2:**
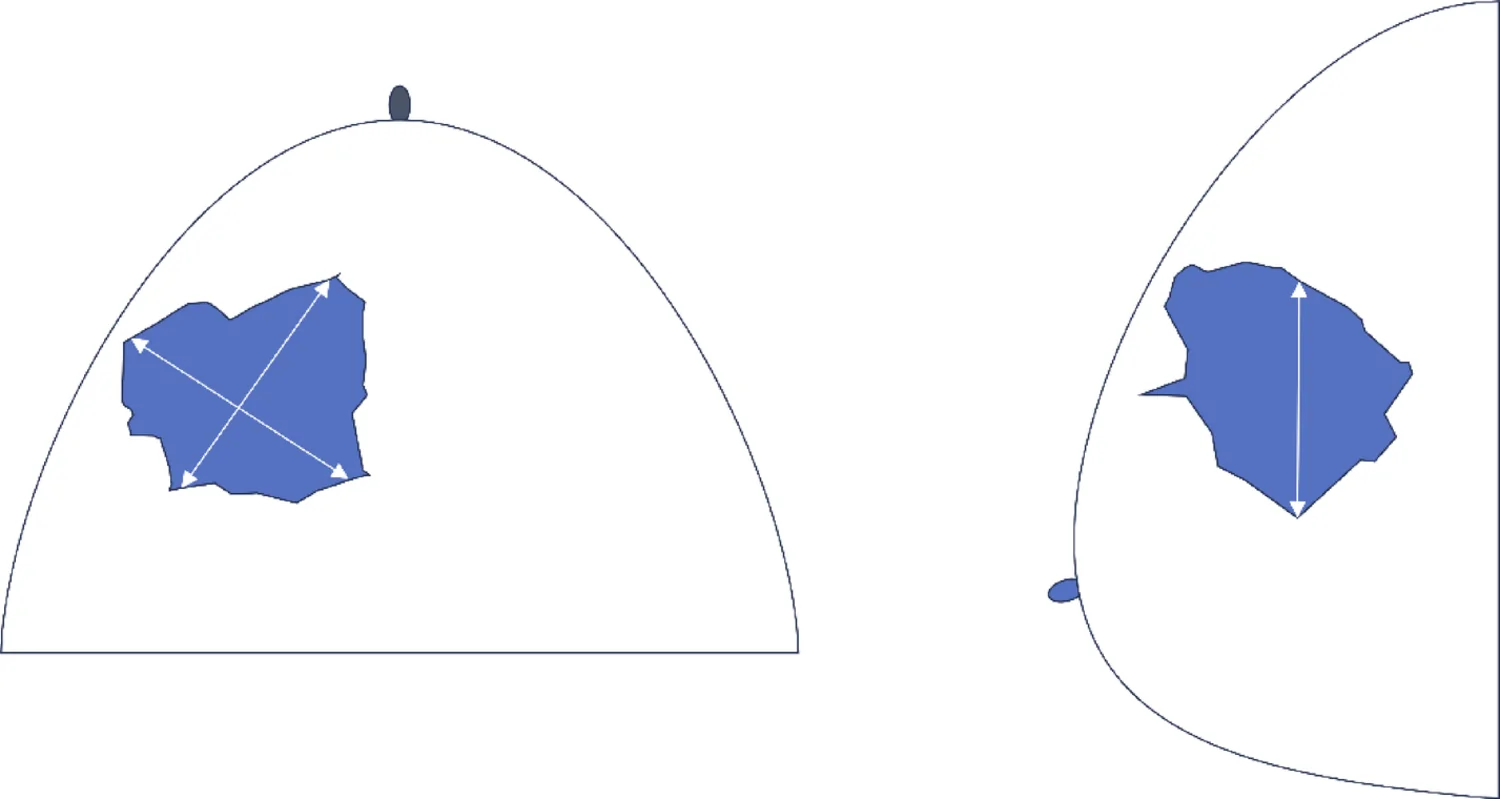
Schematic for describing lesion size

Contrast-enhanced MRI remains the most sensitive modality for assessing the total extent of disease, particularly for identifying an extensive intraductal component [17]. Size should be reported in three dimensions, with the maximum diameter serving as the primary metric for tumor (T)-staging (Fig. 2). Research indicates that MRI provides a more accurate estimation of the final pathological extent versus mammography, ultrasonography, or clinical palpation. Approximately 75% of MRI-detected cancers are within 1.0 cm of their true pathological size [17].

**Fig. 3:**
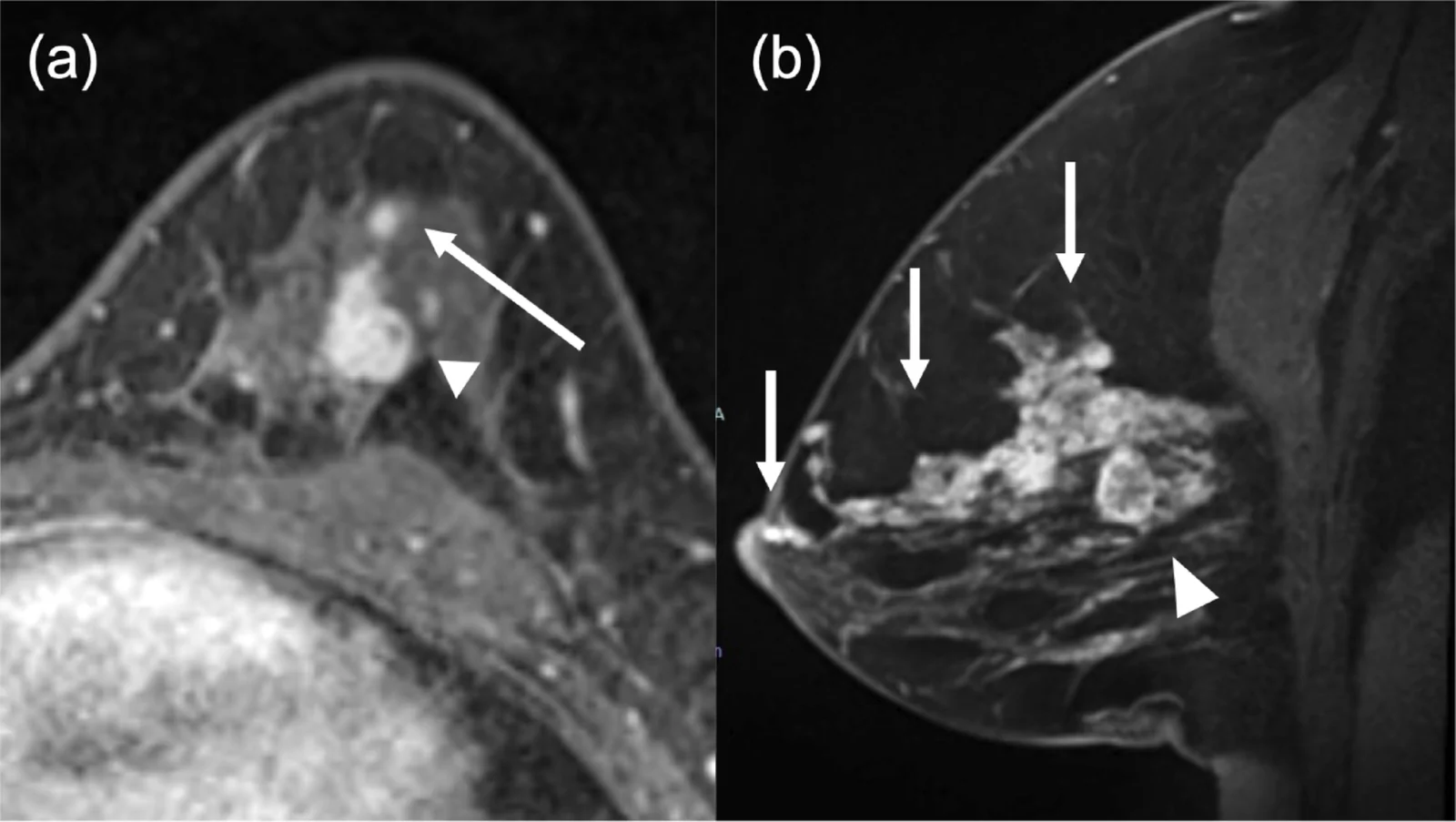
“Additional Close Findings (ACFs)”. **a** Early post-contrast-enhanced T1-weighted image obtained to evaluate the extent of biopsy-proven left breast cancer. An additional 2-mm mass (arrow) located 2 mm anterior to the primary breast cancer (arrowhead) is visible. The extent of the tumor when both lesions are measured together does not exceed the extent of the primary lesion by > 2.0 cm. This second lesion can be identified as an ACF of the primary lesion (category 6). **b** High-resolution contrast-enhanced T1-weighted image (sagittal reconstruction) obtained to evaluate the extent of biopsy-proven right breast cancer. In addition to the primary breast cancer (arrowhead), the non-mass lesion (arrows) has spread extensively and reached the nipple. This lesion requires a separate category (category 5) from that of the primary lesion (category 6)

**Fig. 4:**
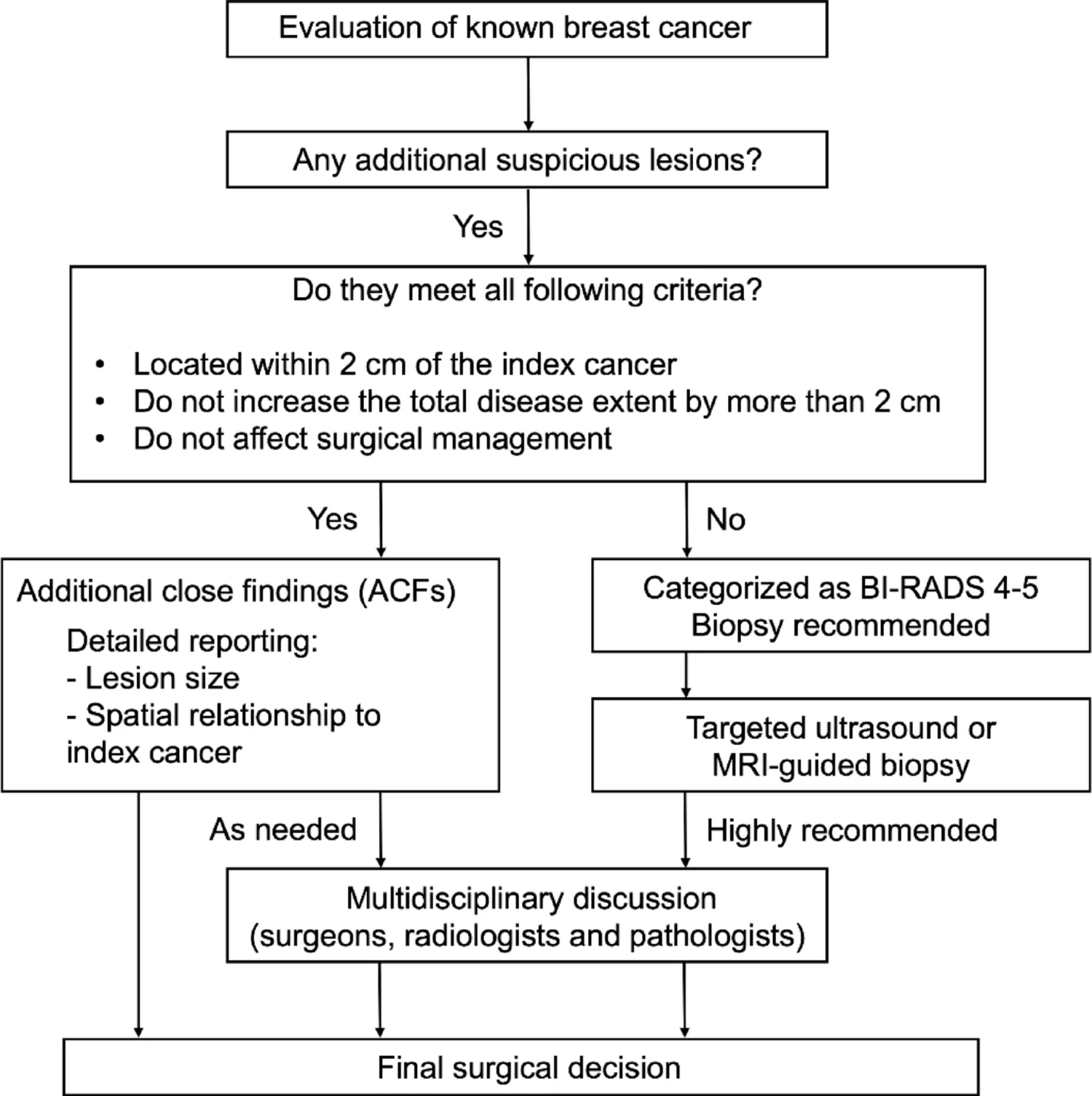
Clinical workflow for additional suspicious lesions in preoperative breast MRI based on BI-RADS v2025. This flowchart illustrates the systematic integration of MRI findings into surgical decision-making. When additional suspicious findings are detected, their management is stratified based on specific criteria. Lesions meeting all criteria are classified as ACFs; these do not routinely require biopsy but should be fully reported with their sizes and spatial relationships relative to the index cancer, with a multidisciplinary discussion between surgeons and radiologists conducted as needed since these lesions are expected to be included within the planned surgical extent. Conversely, lesions that do not meet these criteria are categorized as BI-RADS 4–5, and further evaluation via targeted ultrasound or MRI-guided biopsy is recommended. For these lesions, a multidisciplinary discussion among surgeons, radiologists, and pathologists is highly recommended to carefully evaluate the histopathological results and finalize the optimal surgical approach

**Fig. 5:**
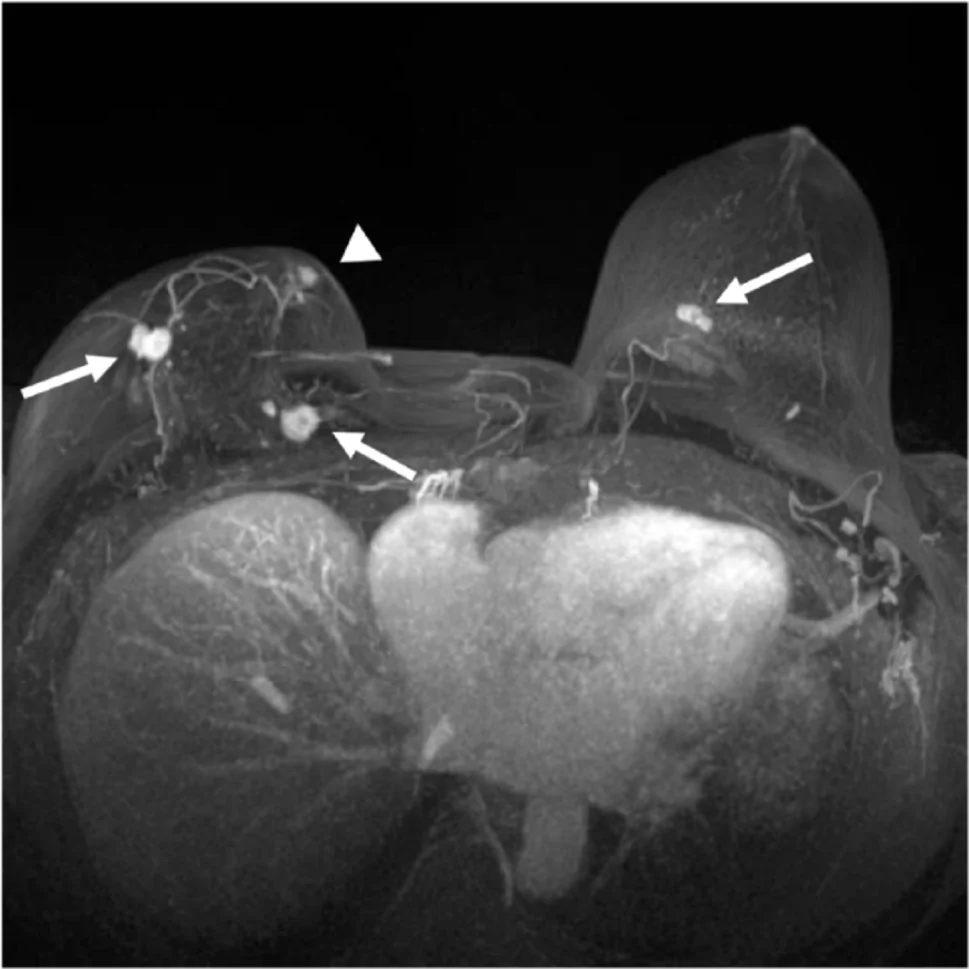
Maximum intensity projection of the early post-contrast T1-weighted image to assess the extent of biopsy-proven multiple breast cancer in the right breast. Two breast masses in the right breast and an additional left breast mass are clearly depicted (arrows). Because of nipple inversion, the contrast effect on the right nipple is prominent (arrowhead)

A critical role of preoperative MRI is the detection of multifocal, multicentric, or contralateral disease that may have been occult on conventional imaging. Abnormal enhancement separate from the primary lesion in the ipsilateral or contralateral breast should be carefully identified because this may change treatment planning. The BI-RADS v2025 update introduced the term “Additional Close Findings” (ACFs) to streamline the management of what were previously known as “satellite” lesions [16]. “ACFs” is defined as suspicious lesions separate from the index cancer (Category 6) but located within 2.0 cm of the index cancer, without increasing the total disease extent by more than 2.0 cm (Fig. 3). Suspicious lesions that fall outside these ACF criteria require separate categorization and often necessitate MRI-detected ultrasonography or MRI-guided biopsy (Fig. 4). Note that the decision on the surgical approach should not be based on preoperative MRI findings alone, given the high false-positive rate of breast MRI [13–15, 18]. Maximum intensity projection of the early post-contrast T1-weighted images is useful for detecting lesions in areas distant from the primary lesion or in the contralateral breast (Fig. 5). Human epidermal growth factor receptor 2 (HER2)-positive breast cancer often presents as multiple lesions, and lobular carcinoma occurs frequently in patients with multiple or bilateral lesions, requiring particular caution [19, 20].

## Tumor-nipple distance

BI-RADS v2025 recommends measuring the distance from the base of the nipple to the center of the findings on the same two-dimensional image or on the maximum intensity projection, with the distance described in whole centimeters when labeling the tumor. When the lesion is close to the nipple, it is also recommended to describe the distance from the nipple to its anterior extent (hereafter defined as tumor-nipple distance [TND]), for surgical planning [16]. This measurement is especially important when considering NSM, which is an increasingly popular choice for its excellent cosmetic outcomes and patient satisfaction. A critical criterion for a safe surgical procedure without tumor recurrence is the exclusion of occult nipple involvement using preoperative MRI. TND on contrast-enhanced MRI is reported to correlate well with the pathological distance [21].

**Fig. 6:**
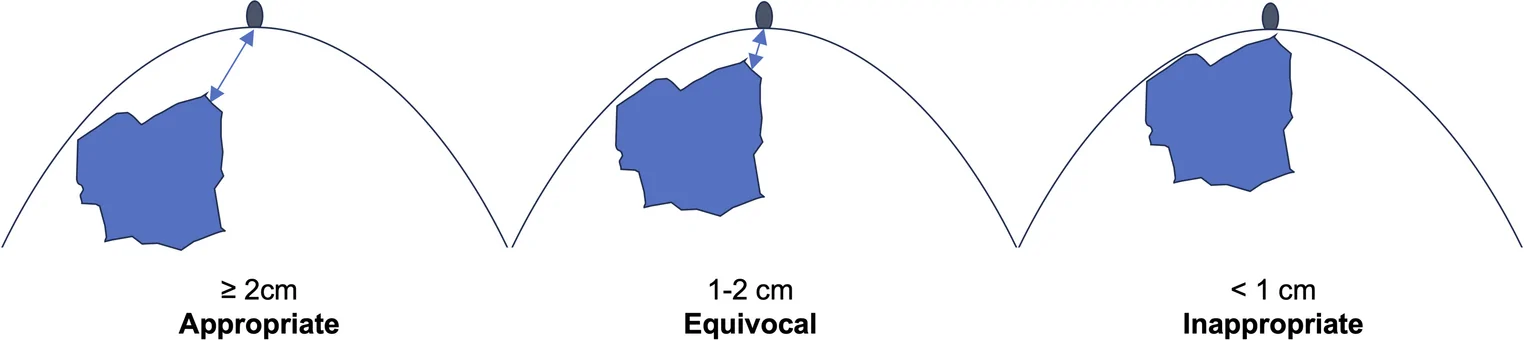
Indications for nipple-sparing mastectomy

**Fig. 7:**
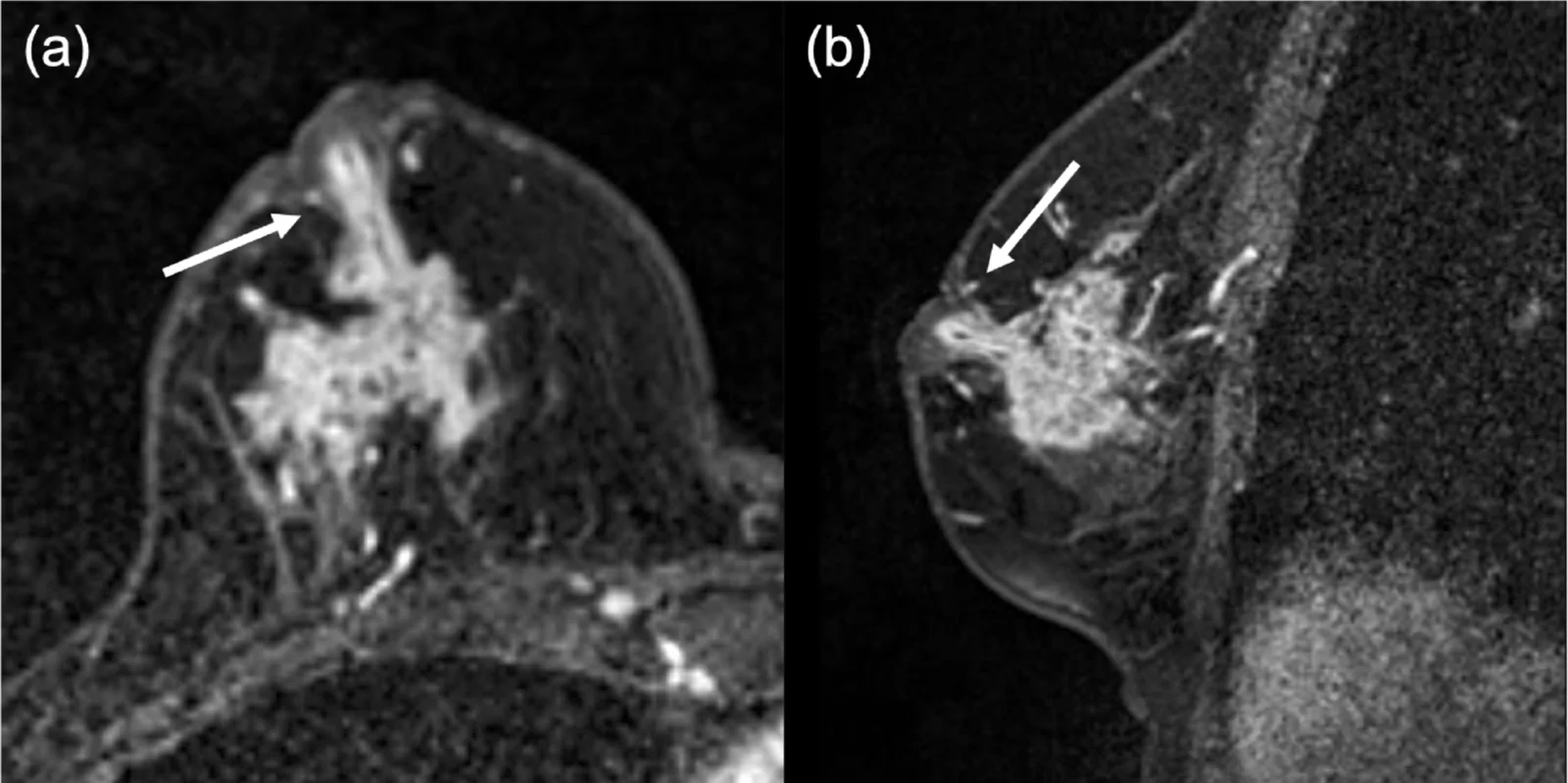
Axial (**a**) and sagittal (**b**) reconstructions of high-resolution contrast-enhanced T1-weighted images obtained to assess the extent of right breast cancer confirmed by preoperative biopsy. Non-mass enhancement extends directly into the nipple, strongly suggesting nipple involvement

**Fig. 8:**
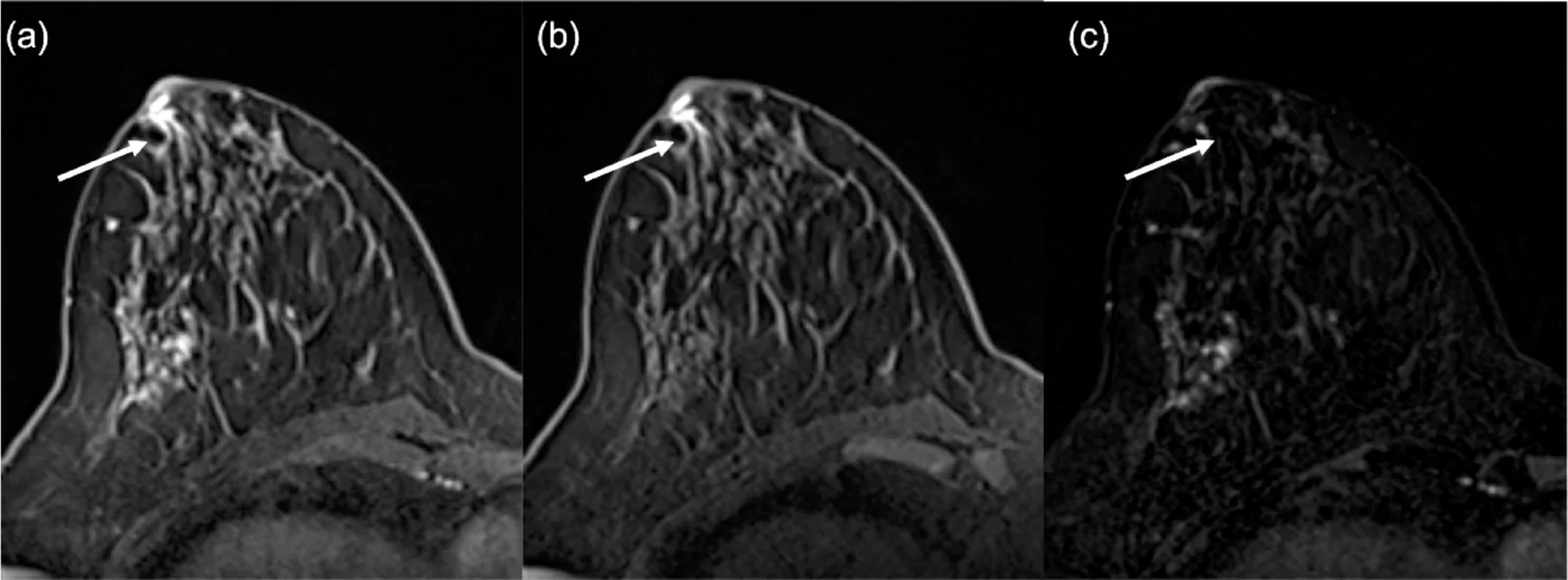
**a** Early post-contrast-enhanced T1-weighted, **b** pre-contrast T1-weighted image, and **c** subtraction image. The high signal directly beneath the nipple (arrows) cannot be determined as a contrast effect based solely on the post-contrast images, but the subtraction images reveal that the signal intensity is not a contrast effect

**Fig. 9:**
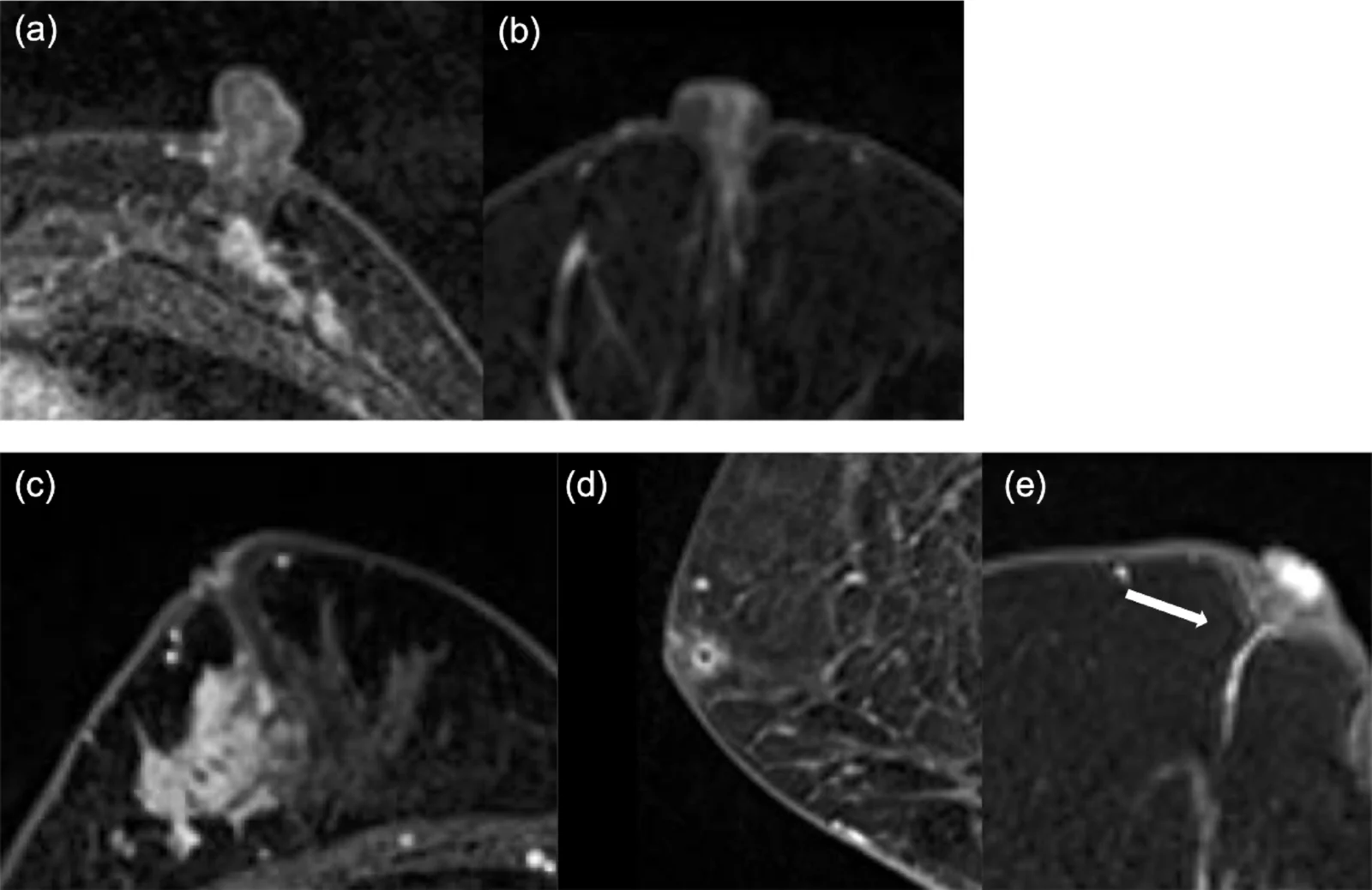
Patchy (**a**) and linear (**b**) enhancement of the normal nipple, inverted nipples (**c** and **d**) and a vessel just below the nipple (**e**)

The TND is a primary predictor of occult nipple involvement. Traditionally, a TND of at least 2.0 cm was required for NSM. However, recent studies suggest that a TND of > 1.0 cm is acceptable in the absence of clinical or radiological nipple involvement (Fig. 6) [22, 23]. Extension of non-mass enhancement (NME) to the nipple base is a strong contraindication for NSM, with a positive predictive value of 86% for pathological nipple involvement (Fig. 7) [21]. In cases with hyperintense signals on pre-contrast T1-weighted images, subtraction of pre-contrast from post-contrast images is useful for the detailed evaluation of subareolar NME (Fig. 8). Radiologists must also distinguish malignancy from physiological nipple enhancement, which typically exhibits three-layered zonal anatomy: superficial linear enhancement at the level of the skin, a nonenhancing zone, and patchy or linear internal nipple enhancement (Fig. 9) [24]. The loss of the normal typically three-layered zonal anatomy of the normal nipple can suggest malignancy [24]. Inverted nipples sometimes mimic masses, and continuity of the superficial linear enhancement may be beneficial for differentiation between the two (Fig. 9). Periareolar vessels may be misdiagnosed as NME, and observing their continuity is useful for diagnosis (Fig. 9) [25].

Beyond direct tumor extension, nipple retraction and nipple-areolar complex skin thickening can be used as secondary morphological changes that are associated with nipple invasion [23].

## Tumor-skin distance

In the context of SSM, preoperative contrast-enhanced MRI is a critical tool for ensuring oncological safety while facilitating immediate breast reconstruction. The primary objective of MRI interpretation for SSM is to accurately predict the pathological skin margin to minimize the risk of local recurrence, which was reported as 3.8% in a recent multicenter study [26]. Unlike ultrasonography, which requires pressing a probe against the skin, which distorts the tissue and the measurable distance, MRI performed in the prone position provides a reproducible measurement of the tumor’s relationship to the skin.

**Fig. 10:**
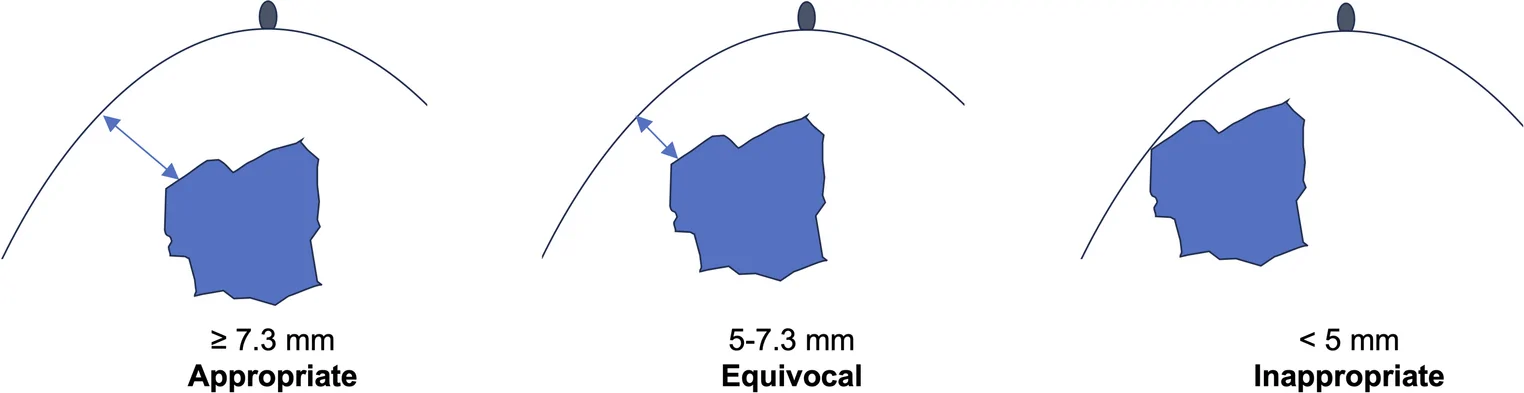
Indications for skin-sparing mastectomy

**Fig. 11:**
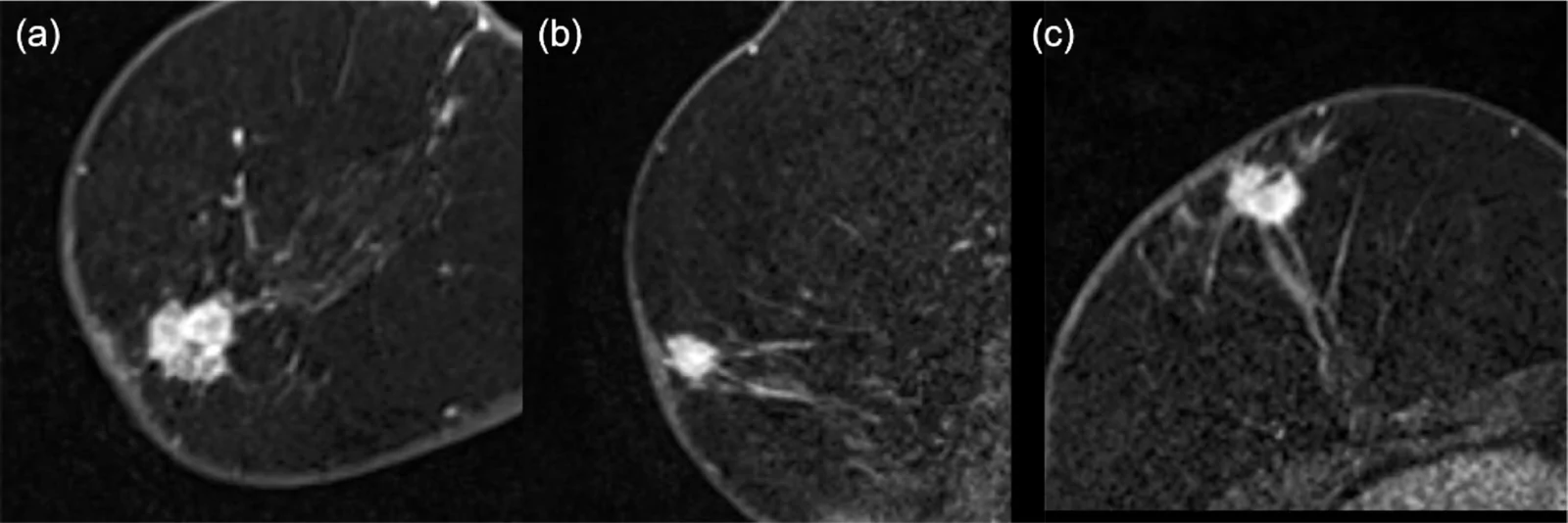
Coronal high-resolution contrast-enhanced T1-weighted image (**a**) and sagittal (**b**) and axial (**c**) reconstruction images of biopsy-proven right breast cancer. The distance between the tumor’s anterior margin and the skin is 2 mm, making preservation of the overlying skin difficult

**Fig. 12:**
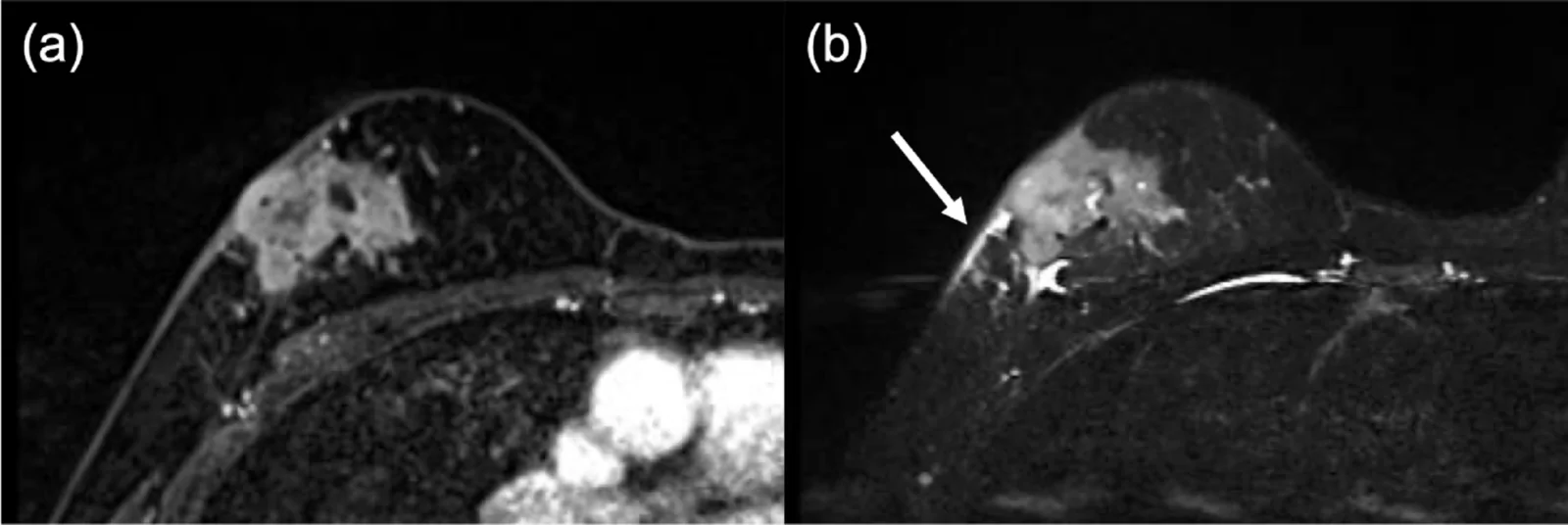
Early post-contrast-enhanced T1-weighted image (**a**) and T2-weighted image (**b**) of biopsy-proven right breast cancer. A large tumor occupying the right breast is widely attached to the skin, depressing and slightly protruding the skin surface. T2-weighted image showing a high-signal area suggesting subcutaneous (arrow) and peritumoral edema. Clinically, skin erythema was also present, raising the suspicion of skin involvement

The most vital metric is the MRI margin, defined as the shortest distance between the tumor edge and the skin surface. Recent Japanese research indicates that an MRI margin of 5 mm may identify pathological margin positivity (sensitivity 54%, specificity 86%) [27]. While an MRI-to-skin distance > 7.3 mm is reported as a clinical criterion for favorable outcomes in SSM, an MRI-to-skin distances < 5 mm is recognized as a threshold linked to a higher risk of residual occult tumor in the skin flap (Figs. 10 and 11). Additionally, the presence of subcutaneous edema on T2-weighted MR images may indicate extensive lymphovascular invasion, potentially precluding conservative mastectomy (Fig. 12) [28].

## Pectoralis muscle involvement

**Fig. 13:**
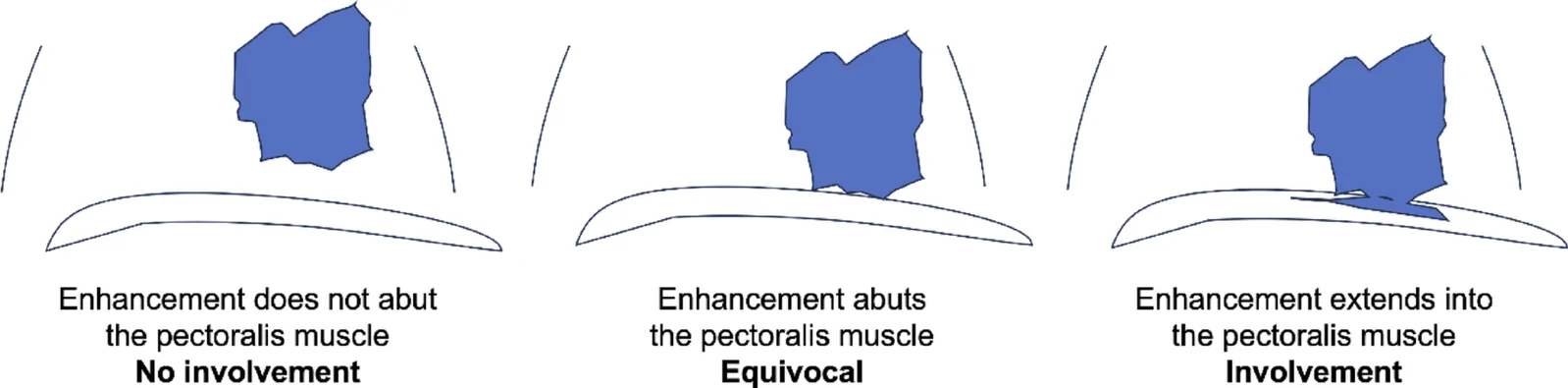
Pectoralis major muscle involvement

**Fig. 14:**
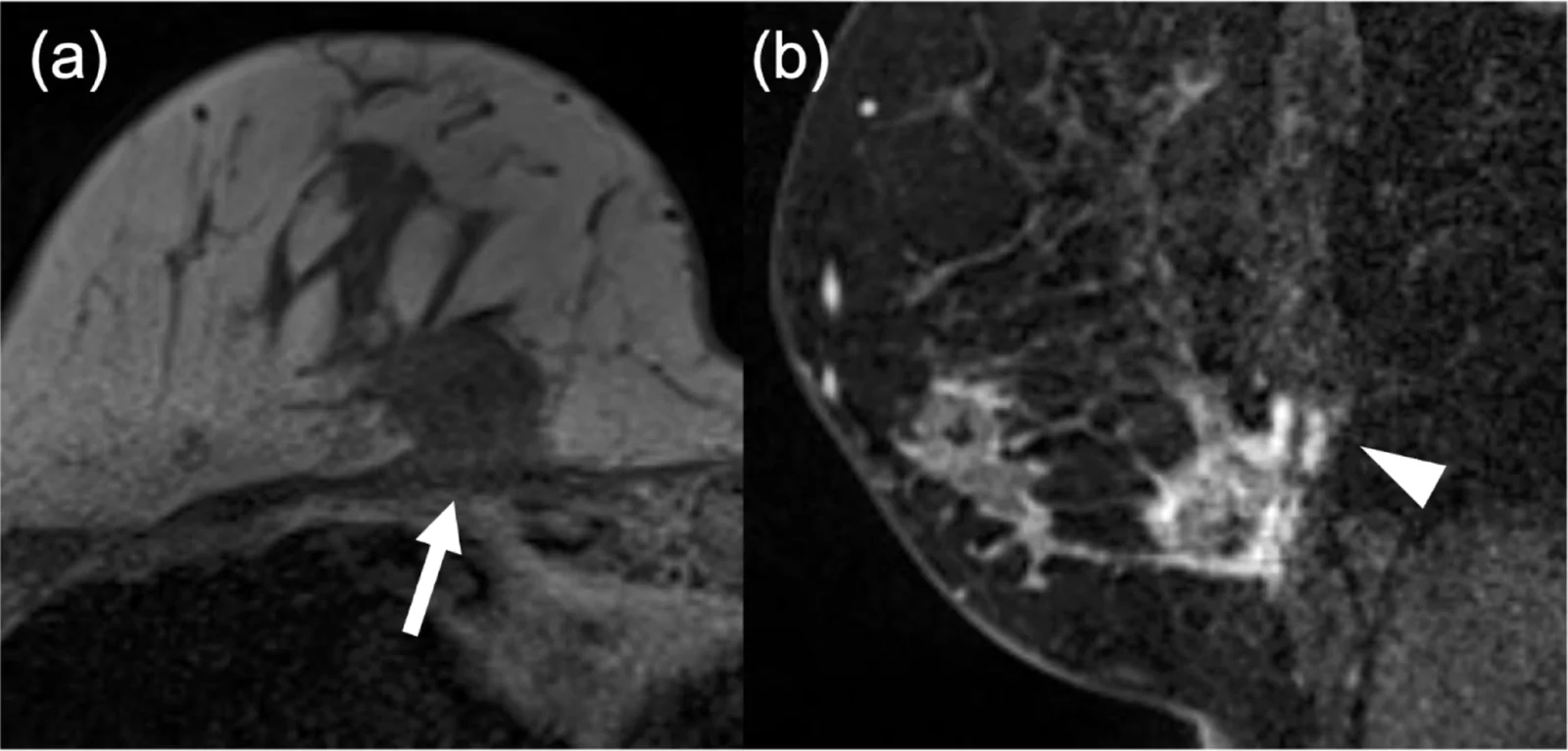
A case of biopsy-proven right breast cancer. Non-contrast T1-weighted image (**a**) showing that a fat signal between the tumor and the pectoralis major muscle is absent, indicating that the tumor is in direct contact with the pectoralis major muscle. The high-resolution contrast-enhanced T1-weighted image (sagittal reconstruction) shows abnormal enhancement within the muscle, strongly suggesting muscle involvement

While muscle involvement (without chest wall invasion) does not alter tumor-node-metastasis (TNM) staging, its presence dictates the necessity of muscle resection. BI-RADS v2025 defines muscle involvement as definitive enhancement extending into the muscle [16]. While abnormal enhancement within the muscle is highly suggestive of definitive involvement [29], invasion can occur in approximately 9.3% of cases without this finding (Figs. 13 and 14) [30]. Notably, obliteration of the retromammary fat plane alone is insufficient for a diagnosis of invasion [16].

Beyond direct enhancement, several secondary MRI features can assist in assessing the risk of pectoralis muscle invasion. Tumors with muscle contour deformation, large overall size, and an extensive area of abutment are more likely to invade the muscle compared with tumors without these features [30]. Invasive cases are more commonly associated with a mass or NME abutting the muscle, whereas tumors that contact the muscle via spicules have a lower degree of suspicion for invasion [30].

## Measurement protocols for TND, skin distance, and pectoralis muscle involvement

To ensure reproducible and accurate measurements, we propose the following protocol based on standard radiological practices. While the early phase typically provides the optimal tumor visualization, spatial assessment should ideally be performed using high-resolution, isotropic sequences that allow for multi-planar reconstruction. If the early-phase acquisition is not isotropic, high-resolution images from subsequent phases may be utilized as a reference for anatomical distance, provided the tumor boundaries remain clearly identifiable. Furthermore, non-fat-suppressed T1-weighted imaging may be useful for assessing the preservation or obliteration of the fat signal in the retromammary space.

## Lymph nodes

**Fig. 15:**
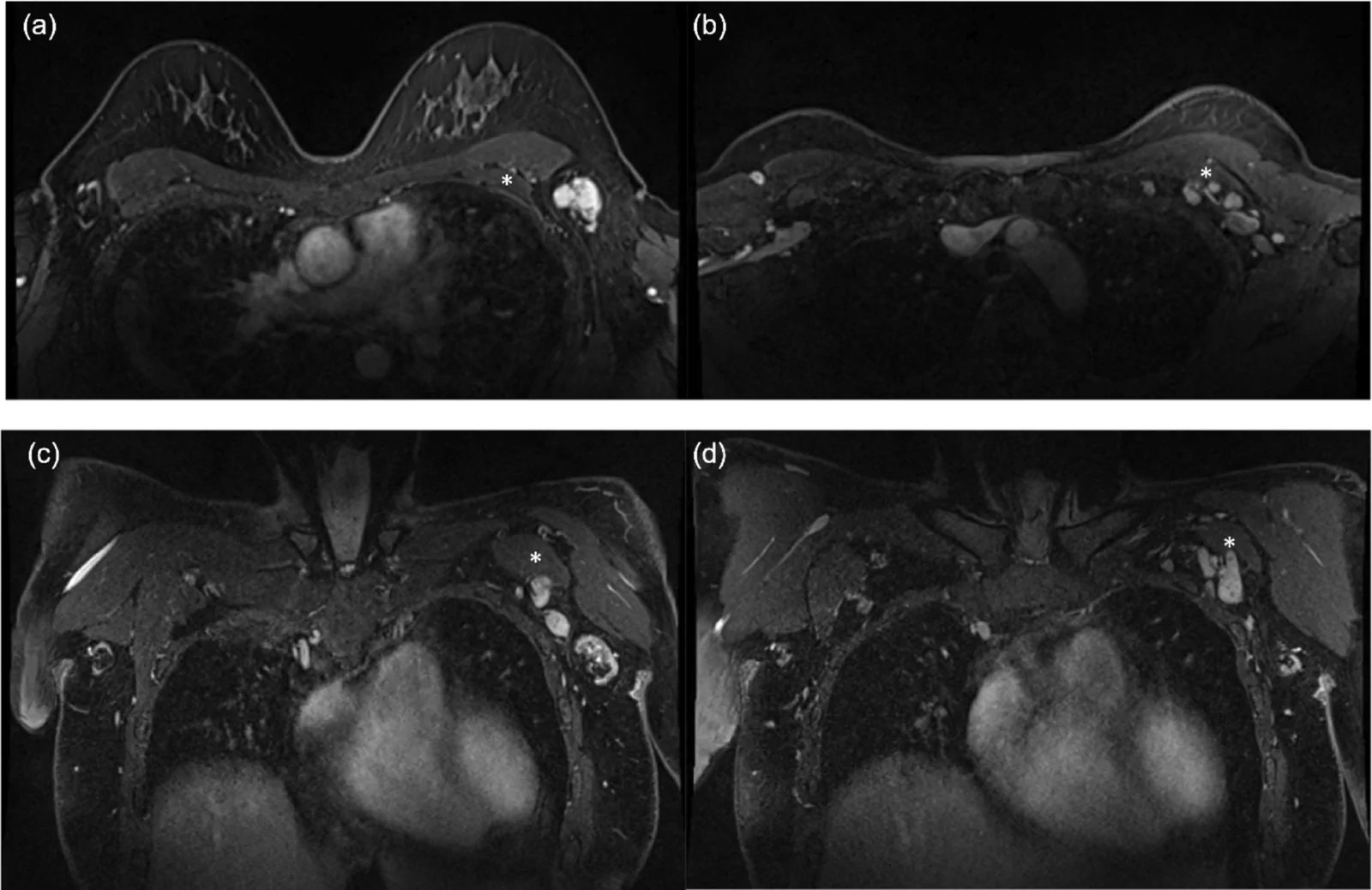
Classification and evaluation of axillary lymph nodes. Axillary lymph nodes are classified into level I–III relative to the pectoralis minor muscle. **a**, **b** Axial and **c**, **d** coronal fat-suppressed contrast-enhanced MR images. Abnormal left axillary lymph nodes are observed in **a** level I, **b**, **c** level I and II, and **d** level II and III. The asterisk (*) indicates the pectoralis minor muscle

Detecting lymph node metastases before treatment is indispensable for planning surgery and radiation therapy. In an important revision, BI-RADS v2025 has designated lymph node evaluation as a distinct section [16]. The lymph nodes requiring assessment on breast MRI are intramammary, axillary, and internal mammary lymph nodes. There are no strict size criteria, but subjectively abnormal nodes should be described with their location or level of the axilla. The level of axillary lymph nodes is defined as follows relative to the pectoralis minor muscle: those lateral to its outer border are classed as Level I, those anterior (interpectoral or Rotter’s lymph node) and posterior to the muscle as Level II, and those medial to the muscle’s inner border (infraclavicular) as Level III (Fig. 15). The number of axillary nodes is important for treatment planning. For the internal mammary lymph nodes, it is desirable to describe the side and the intercostal space number.

**Fig. 16:**
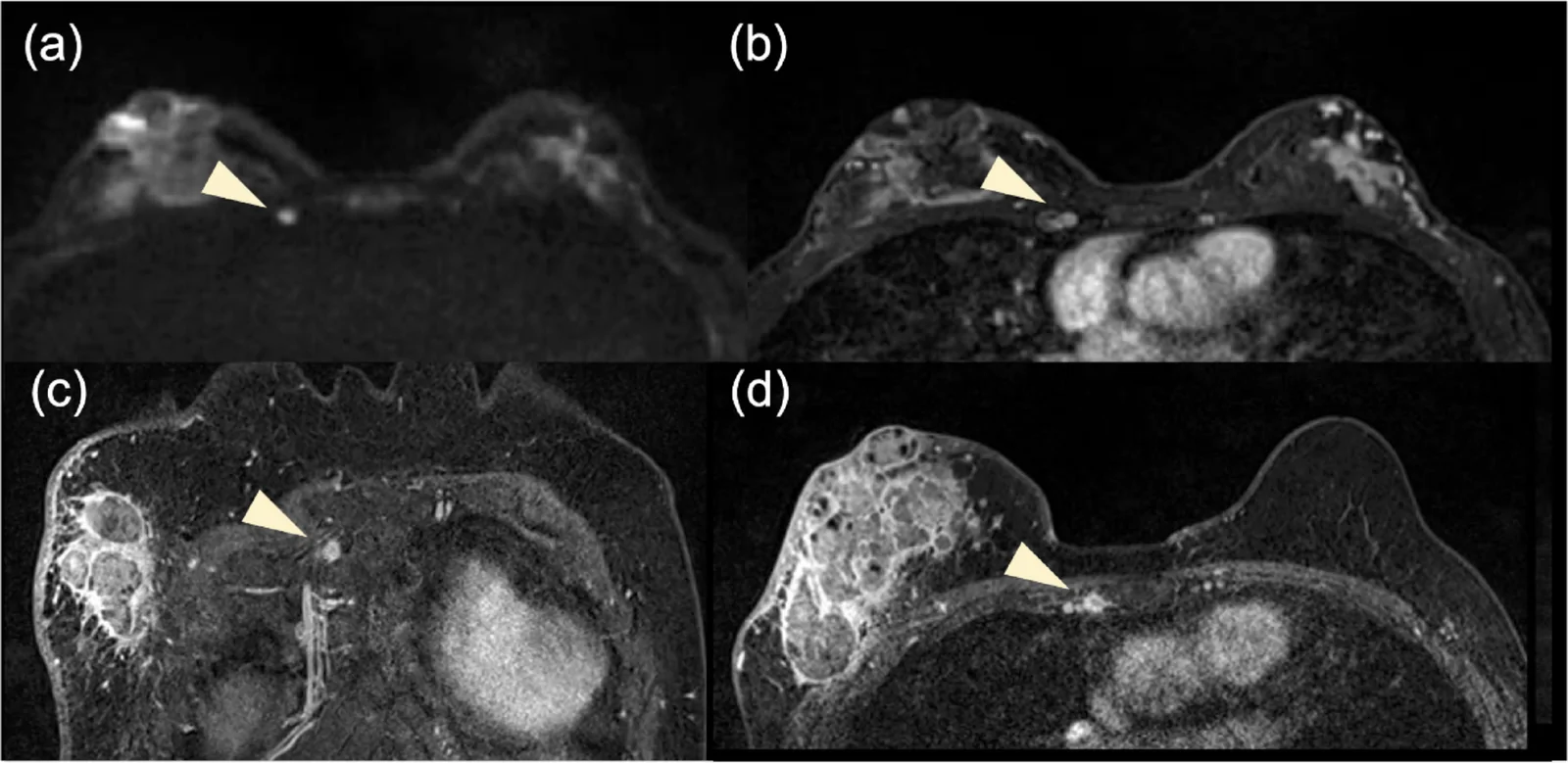
Abnormal findings in internal mammary lymph node imaging. An abnormal right internal mammary lymph node was easier to detect on diffusion-weighted imaging (**a**) than on the early post-contrast T1-weighted image (**b**). In another case, an abnormal right internal mammary lymph node was easier to detect on coronal high-resolution contrast-enhanced T1-weighted imaging (**c**) compared with the early post-contrast T1-weighted image (**d**)

Diffusion-weighted images are useful for detecting internal mammary lymph node metastases [31]. Coronal contrast-enhanced MRI is also useful for distinguishing internal mammary lymph nodes from adjacent internal mammary vessels (Fig. 16).

## Radiofrequency ablation

Radiofrequency ablation (RFA) is emerging as a minimally invasive local therapy for early-stage breast cancer, offering an alternative to conventional surgical resection by inducing coagulative necrosis while preserving surrounding healthy tissue [32, 33]. In Japan, RFA received official public health insurance coverage in December 2023 [34], with eligibility criteria strictly following the national guidelines [35] and the protocol of the Phase III RAFAELO (Radiofrequency Ablation Therapy for Early Breast Cancer as Local therapy) trial. These criteria require the lesion to be a single localized invasive or noninvasive ductal carcinoma ≤ 1.5 cm on all preoperative imaging, including contrast-enhanced MRI, without skin invasion or clinical lymph node metastasis [36]. Internationally, the 2023 Kyoto Breast Cancer Consensus Conference proposed even more stringent indications, recommending RFA primarily for well-localized, luminal A-like tumors ≤ 1.0 cm in clinically node-negative patients, while most previous studies included tumors up to 2.0 cm [32, 33].

Meta-analyses of thermal ablation for breast cancers ≤ 2.0 cm have reported a complete ablation rate of 92% and local recurrence rates ranging from 0% to 3% over a median follow-up of 15–61 months [37]. Another systematic review of 17 studies (399 cases) further demonstrated a technical success rate of approximately 99%, a complete ablation rate of 96%, and a low complication rate of 6.80%, with skin burns being the most frequent adverse event (2.02%) [38]. A significantly higher recurrence rate in tumors > 2.0 cm compared to those ≤ 2.0 cm (10% vs. 2.3%; *p* = 0.015) was shown in a retrospective study [36], justifying the selection of lesions ≤ 2.0 cm as the optimal candidates for RFA. The clinical efficacy of RFA has been robustly demonstrated in the RAFAELO study [39]. Among 370 patients who underwent RFA, 353 completed a 5-year follow-up, yielding a 5-year ipsilateral breast tumor-free survival rate of 98.6% (90% CI 97.1–99.3%). This result confirmed the non-inferiority of RFA to historical controls of partial mastectomy. Safety outcomes were excellent, with only two recurrences and a single case of grade 3 skin ulceration reported. Consequently, MRI interpretation is essential to determine the size and exact location of tumor center, and to rule out extensive intraductal components or suspected multifocality, both of which remain absolute contraindications for the procedure.

## Conclusion

This review primarily focuses on the preoperative assessment of patients undergoing primary surgery, while MRI is also crucial for evaluating the response to neoadjuvant systemic therapy. The integration of preoperative MRI into breast cancer management has moved beyond simple detection toward providing a detailed anatomical roadmap for complex oncoplastic surgery. The BI-RADS v2025 updates provide much-needed standardization, particularly through the introduction of the ACFs and specific guidelines for lymph node assessment. The optimal use of preoperative MRI depends on a multidisciplinary approach in which radiologists provide standardized, surgically-relevant metrics that allow surgeons to maximize cosmetic outcomes through NSM and SSM without compromising oncological safety.

## References

[1] Plana MN, Carreira C, Muriel A, Chiva M, Abraira V, Emparanza JI, et al. Magnetic resonance imaging in the preoperative assessment of patients with primary breast cancer: systematic review of diagnostic accuracy and meta-analysis. Eur Radiol. 2012;22:26–38. 10.1007/s00330-011-2238-8. 21847541

[2] Dontchos BN, Phelps MD, Rahbar H, Lam DL. Pre-treatment breast MRI: clinical indications, outcomes, and future directions. J Magn Reson Imaging. 2025. 10.1002/jmri.29741. 39953849

[3] Turnbull LW, Brown SR, Olivier C, Harvey I, Brown J, Drew P, et al. Multicentre randomised controlled trial examining the cost-effectiveness of contrast-enhanced high field magnetic resonance imaging in women with primary breast cancer scheduled for wide local excision (COMICE). Health Technol Assess. 2010;14:1–182. 10.3310/hta14010. 20025837

[4] Peters NHGM, van Esser S, van den Bosch MAAJ, Storm RK, Plaisier PW, van Dalen T, et al. Preoperative MRI and surgical management in patients with nonpalpable breast cancer: the MONET—randomised controlled trial. Eur J Cancer. 2011;47:879–86. 10.1016/j.ejca.2010.11.035. 21195605

[5] Balleyguier C, Dunant A, Ceugnart L, Kandel M, Chauvet M-P, Chérel P, et al. Preoperative breast magnetic resonance imaging in women with local ductal carcinoma in situ to optimize surgical outcomes: results from the randomized phase III trial IRCIS. J Clin Oncol. 2019;37:885–92. 10.1200/JCO.18.00595. 30811290

[6] Gonzalez V, Sandelin K, Karlsson A, Åberg W, Löfgren L, Iliescu G, et al. Preoperative MRI of the breast (POMB) influences primary treatment in breast cancer: a prospective, randomized, multicenter study. World J Surg. 2014;38:1685–93. 10.1007/s00268-014-2605-0. 24817517

[7] Gonzalez V, Arver B, Löfgren L, Bergkvist L, Sandelin K, Eriksson S. Impact of preoperative breast MRI on 10-year survival of patients included in the Swedish randomized multicentre POMB trial. BJS Open. 2021;5:zrab088. 10.1093/bjsopen/zrab088. PMC8493003 34611701

[8] Sardanelli F, Trimboli RM, Houssami N, Gilbert FJ, Helbich TH, Álvarez Benito M, et al. Magnetic resonance imaging before breast cancer surgery: results of an observational multicenter international prospective analysis (MIPA). Eur Radiol. 2022;32:1611–23. 10.1007/s00330-021-08240-x. PMC8831264 34643778

[9] Kuhl CK, Strobel K, Bieling H, Wardelmann E, Kuhn W, Maass N, et al. Impact of preoperative breast MR imaging and MR-guided surgery on diagnosis and surgical outcome of women with invasive breast cancer with and without DCIS component. Radiology. 2017;284:645–55. 10.1148/radiol.2017161449. 28445683

[10] Ha SM, Chae EY, Cha JH, Kim HH, Shin HJ, Choi WJ. Breast MR imaging before surgery: outcomes in patients with invasive lobular carcinoma by using propensity score matching. Radiology. 2018;287:771–7. 10.1148/radiol.2018171472. 29388904

[11] Cozzi A, Di Leo G, Houssami N, Gilbert FJ, Helbich TH, Álvarez Benito M, et al Preoperative breast MRI reduces reoperations for unilateral invasive lobular carcinoma: a patient-matched analysis from the MIPA study. Eur Radiol. 2025. . 10.1007/s00330-024-11338-7. 40016317

[12] Cozzi A, Di Leo G, Houssami N, Gilbert FJ, Helbich TH, Álvarez Benito M, et al. Preoperative breast MRI positively impacts surgical outcomes of needle biopsy-diagnosed pure DCIS: a patient-matched analysis from the MIPA study. Eur Radiol. 2023. 10.1007/s00330-023-10409-5. PMC11166778 37999727

[13] Gradishar WJ, Chew H, Rugo HS, Dang C, Santa-Maria CA, Moran MS et al. NCCN Clinical Practice Guidelines in Oncology (NCCN Guidelines^®^), Breast Cancer Version 2.2026. National comprehensive cancer network; 2026. Available from: https://www.nccn.org/professionals/physician_gls/pdf/breast.pdf [Accessed April 11, 2026].

[14] American Society of Breast Surgeons. Diagnostic and screening magnetic resonance imaging of the breast [Internet]. American Society of Breast Surgeons. 2018. Available from: https://www.breastsurgeons.org/docs/statements/Consensus-Guideline-on-Diagnostic-and-Screening-Magnetic-Resonance-Imaging-of-the-Breast.pdf

[15] Mann RM, Kuhl CK, Kinkel K, Boetes C, Breast MRI. Guidelines from the European society of breast imaging. Eur Radiol. 2008;18:1307–18. 10.1007/s00330-008-0863-7. PMC2441490 18389253

[16] DeMartini WB, Strigel RM, Pinker K, Rahbar H, Wang LC. Magnetic resonance imaging. ACR BI-RADS v2025 manual. Reston, VA: American College of Radiology; 2025.

[17] Mann RM, Cho N, Moy L, Breast MRI. State of the art. Radiology. 2019;292:520–36. 10.1148/radiol.2019182947. 31361209

[18] Kubota K, Nakashima K, Nakashima K, Kataoka M, Inoue K, Goto M, et al. The Japanese breast cancer society clinical practice guidelines for breast cancer screening and diagnosis, 2022 edition. Breast Cancer. 2024;31:157–64. 10.1007/s12282-023-01521-x. PMC10901949 37973686

[19] Poenaru M-O, Amza M, Toma C-V, Augustin F-E, Pacu I, Zampieri G, et al. Multicentric and multifocal breast tumors-narrative literature review. Cancers (Basel). 2025;17:3380. 10.3390/cancers17203380. PMC12564034 41154435

[20] Schulze AK, Hoskin TL, Moldoveanu D, Sturz JL, Boughey JC. Tumor characteristics of bilateral breast cancer compared with unilateral breast cancer. Ann Surg Oncol. 2024;31:947–56. 10.1245/s10434-023-14451-x. 37906382

[21] Bae SJ, Cha YJ, Eun NL, Ji JH, Kim D, Lee J, et al Diagnostic accuracy of nonmass enhancement at breast MRI in predicting tumor involvement of the nipple: a prospective study in a single institution. Radiology. 2021. 10.1148/radiol.2021204136. 34254854

[22] Mariscotti G, Durando M, Houssami N, Berzovini CM, Esposito F, Fasciano M, et al. Preoperative MRI evaluation of lesion-nipple distance in breast cancer patients: thresholds for predicting occult nipple-areola complex involvement. Clin Radiol. 2018;73:735–43. 10.1016/j.crad.2018.03.008. 29678274

[23] Lee SC, Mendez-Broomberg K, Eacobacci K, Vincoff NS, Gupta E, McElligott SE. Nipple-sparing mastectomy: What the radiologist should know. Radiographics. 2022;42:321–39. 10.1148/rg.210136. 35179983

[24] Gao Y, Brachtel EF, Hernandez O, Heller SL. An analysis of nipple enhancement at breast MRI with radiologic-pathologic correlation. Radiographics. 2019;39:10–27. 10.1148/rg.2019180039. 30547729

[25] Nakajima H, Imanishi N, Aiso S. Arterial anatomy of the nipple-areola complex. Plast Reconstr Surg. 1995;96:843–5. 10.1097/00006534-199509001-00012. 7652057

[26] Ogiya A, Nagura N, Shimo A, Nogi H, Narui K, Seki H, et al. Long-term outcomes of breast cancer patients with local recurrence after mastectomy undergoing immediate breast reconstruction: A retrospective multi-institutional study of 4153 cases. Ann Surg Oncol. 2023;30:6532–40. 10.1245/s10434-023-13832-6. 37405666

[27] Kataoka A, Sawaki M, Okumura S, Onishi S, Iwase M, Sugino K, et al. Prediction of pathological margin status using preoperative contrast-enhanced MRI in patients with early breast cancer who underwent skin-sparing mastectomy. Breast J. 2019;25:202–6. 10.1111/tbj.13194. 30697867

[28] Uematsu T. Focal breast edema associated with malignancy on T2-weighted images of breast MRI: peritumoral edema, prepectoral edema, and subcutaneous edema. Breast Cancer. 2015;22:66–70. 10.1007/s12282-014-0572-9. 25336185

[29] Morris EA, Schwartz LH, Drotman MB, Kim SJ, Tan LK, Liberman L, et al. Evaluation of pectoralis major muscle in patients with posterior breast tumors on breast MR images: early experience. Radiology. 2000;214:67–72. 10.1148/radiology.214.1.r00ja1667. 10644103

[30] Myers KS, Stern E, Ambinder EB, Oluyemi ET. Breast cancer abutting the pectoralis major muscle on breast MRI: what are the clinical implications? Br J Radiol. 2021;94:20201202. 10.1259/bjr.20201202. PMC8011236 33353392

[31] Harada TL, Uematsu T, Nakashima K, Sugino T, Nishimura S, Hayashi T, et al. A preliminary study on diffusion-weighted imaging with background suppression and maximum intensity projection for screening internal mammary lymph node metastasis of breast cancer. Acta Radiol Open. 2025;14:20584601251339017. 10.1177/20584601251339017. PMC12163280 40519659

[32] Toi M, Kinoshita T, Benson JR, Jatoi I, Kataoka M, Han W, et al. Non-surgical ablation for breast cancer: an emerging therapeutic option. Lancet Oncol. 2024;25:e114–25. 10.1016/S1470-2045(23)00615-0. 38423057

[33] Takayama S, Murata T, Watase C, Maeda H, Nakashoji A, Ogawa A, et al Emerging advances in non-surgical ablation for early-stage breast cancer. Jpn J Radiol. 2026. . 10.1007/s11604-026-01951-5. 41642532

[34] Ministry of Health, Labour and Welfare. [Partial revision of the points to note in implementation accompanying the partial revision of the calculation method of medical fees]. 2023. Available from: https://www.mhlw.go.jp/content/12404000/001173326.pdf (in Japanese). Accessed 11 April 2026.

[35] The Japan Breast Cancer Society. [Guidelines for the proper use of radiofrequency ablation (RFA) for breast cancer]. 2023. Available from: https://www.jbcs.gr.jp/uploads/files/shikaku%20elearning/RFA/rfa.20231117.pdf (in Japanese) . Accessed 11 April 2026.

[36] Ito T, Oura S, Nagamine S, Takahashi M, Yamamoto N, Yamamichi N, et al. Radiofrequency ablation of breast cancer: a retrospective study. Clin Breast Cancer. 2018;18:e495–500. 10.1016/j.clbc.2017.09.007. 29079443

[37] van de Voort EMF, Struik GM, Birnie E, Moelker A, Verhoef C, Klem TMAL. Thermal ablation as an alternative for surgical resection of small (≤ 2 cm) breast cancers: a meta-analysis. Clin Breast Cancer. 2021;21:e715–30. 10.1016/j.clbc.2021.03.004. 33840627

[38] Xia L-Y, Hu Q-L, Xu W-Y. Efficacy and safety of radiofrequency ablation for breast cancer smaller than 2 cm: a systematic review and meta-analysis. Front Oncol. 2021;11:651646. 10.3389/fonc.2021.651646. PMC8126716 34012918

[39] Kinoshita T, Takayama S, Fujisawa T, Watanabe K, Yamamoto N, Shien T, et al. Radiofrequency ablation for early stage breast cancer as a potential alternative to partial mastectomy: 5-year results from the multiple-center, single-arm, phase 3 RAFAELO study (NCCH1409). Ann Surg Oncol. 2026. 10.1245/s10434-026-19220-0. 41708933

